# Robust discovery of mutational signatures using power posteriors

**DOI:** 10.1371/journal.pcbi.1014372

**Published:** 2026-06-11

**Authors:** Catherine Xue, Jeffrey W. Miller, Scott L. Carter, Jonathan H. Huggins

**Affiliations:** 1 Department of Biostatistics, Harvard University, Boston, Massachusetts, United States of America; 2 Department of Data Science, Dana–Farber Cancer Institute, Boston, Massachusetts, United States of America; 3 Department of Mathematics & Statistics, Boston University, Boston, Massachusetts, United States of America; 4 Faculty of Computing & Data Science, Boston University, Boston, Massachusetts, United States of America; Virginia Polytechnic Institute and State University, UNITED STATES OF AMERICA

## Abstract

Mutational processes, such as the molecular effects of carcinogenic agents or defective DNA repair mechanisms, produce different mutation types with characteristic frequency profiles, known as mutational signatures. Non-negative matrix factorization (NMF) has been successfully used to discover many mutational signatures, yielding novel insights into cancer etiology and informing targeted therapies. However, the NMF model is only a rough approximation to reality, and even small departures from this assumed model can have large negative effects on the accuracy and reliability of the results. We propose *BayesPowerNMF*, a Bayesian NMF method that provides nonparametric robustness to model misspecification, principled automated selection of the number of latent processes, and uncertainty quantification of model parameters. In extensive simulation studies, we find that our proposed approach recovers more true signatures with greater accuracy than current leading methods. On whole-genome sequencing data for six cancer types from the ICGC/TCGA Pan-Cancer Analysis of Whole Genomes Consortium, we find that our method is able to accurately recover more signatures than the current state-of-the-art.

## Author summary

The genomes of cancerous cells typically contain thousands to tens of thousands of mutations. These mutations arise from a variety of DNA-damaging processes, such as ultraviolet radiation from the sun, chemicals in cigarette smoke, and broken DNA repair mechanisms within the cells. Each of these processes creates distinctive patterns of mutation types, known as mutational signatures. Discovering and characterizing these mutational signatures can provide scientific insight into how tumors develop and yield clinical value by helping guide the use of targeted therapies. However, current approaches for analyzing signatures rely on simplifying assumptions that do not hold in practice. Therefore, they can produce incorrect conclusions about the existence of mutational process and the activity level of real processes. We introduce a statistical method that is designed to work even when the modeling assumptions are not exactly correct. Additionally, our approach automatically determines how many underlying mutational processes are supported by the data and reports uncertainty in the inferred signatures and their activity levels. In large simulation studies, our approach recovers more true signatures more accurately than leading tools. Applied to tumor genome data from six cancer types, it recovers more matches to previously established signatures while maintaining high accuracy.

## Introduction

Carcinogenic processes such as UV radiation, smoking, defective DNA repair mechanisms, and naturally occurring biochemical reactions generate characteristic patterns of somatic mutations known as mutational signatures [1–3]. While these processes cannot be observed directly in patients, the cumulative effect of multiple processes on an individual tumor can be quantified using genome sequencing, and their distinct mutational signatures can be inferred using statistical modeling. Non-negative matrix factorization (NMF) models have proven effective in estimating mutational signatures as well as the mutational load due to each signature in each tumor sample [4,5]. Mutational signatures analysis has contributed to novel insights in a variety of areas of cancer research [1,6–9] and has emerging translations to clinical outcomes [10,11].

Existing methods for mutational signatures analysis are fundamentally limited by the fact that they assume—either explicitly or implicitly—a particular probabilistic model for how mutations arise. However, any assumed model will only be a rough approximation to reality. Unfortunately, using an incorrect model—known as *model misspecification*—can lead to spurious inferences [12–15]. In particular, a key challenge in mutational signature discovery is determining the number of active signatures [5,6,16,17], and standard statistical methods for this type of model selection problem tend to be extremely sensitive to misspecification [12,15]. For instance, methods based on automatic relevance determination (ARD) [16,18] and Bayesian spike-and-slab models [19] tend to overestimate the number of signatures when there is mild overdispersion [20]. Of course, robustness to outliers can be obtained using overdispersed models such as negative binomial [21,22], but this does not handle any other types of misspecification. Perhaps due to this sensitivity, the current leading methods rely heavily on *ad hoc* techniques such as manual filtering [8] or neural networks [23] for determining the number of signatures.

**Fig 1:**
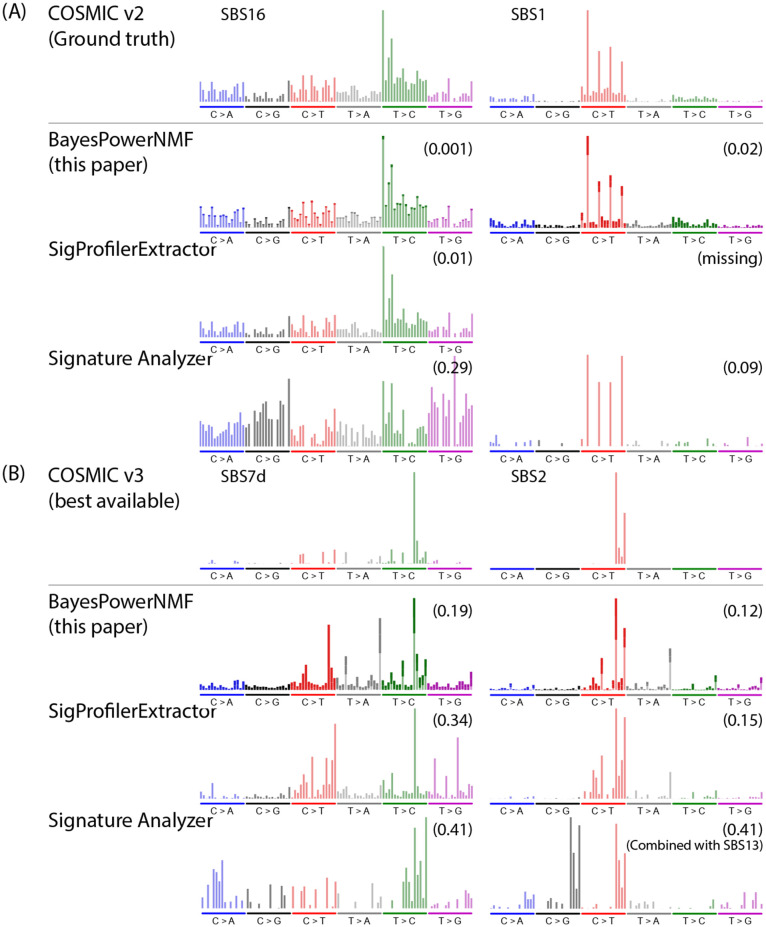
BayesPowerNMF finds more true processes and fewer spurious processes than competitors. ***(A)*** Examples from simulated misspecified liver cancer data. For two ground truth signatures used to generate the data (SBS16 and SBS1 from COSMIC v2, shown in the top row), we show the best-matching signatures inferred by two leading methods (SigProfilerExtractor and SignatureAnalyzer) and our method (BayesPowerNMF). The cosine errors between inferred and true signatures are in parentheses. When the model is misspecified, SigProfilerExtractor tends to miss signatures (such as SBS1 in this case) and SignatureAnalyzer tends to have significantly higher cosine errors compared to BayesPowerNMF. ***(B)*** Examples from real melanoma data. Same as in **(A)**, but comparing with the closest match to SBS7d and SBS2 from COSMIC v3. For BayesPowerNMF, the bolded section of each bar indicates a 95% credible interval, quantifying the uncertainty in each signature. Meanwhile, SigProfilerExtractor and SignatureAnalyzer only provide point estimates that may be misleading in cases such as SBS7d for which there is high uncertainty. See Fig C.1 in S1 Appendix for SigMoS, which is similar to SigProfilerExtractor, with higher cosine error except for SBS1 on the liver data.

In this paper, we show that leading methods for mutational signatures analysis are not robust to model misspecification, and we introduce a novel method that exhibits better performance under misspecification. Specifically, we investigate the degree to which misspecification causes existing methods to (1) fail to find important processes or (2) infer spurious processes that do not actually exist. Our findings indicate a lack of robustness in two widely used methods: SigProfilerExtractor [5,8,24] and SignatureAnalyzer [8,16,18]; see Fig 1 for examples. We also compare against SigMoS [22], which explictly models overdispersion in mutation counts; however, it similarly fails to address more general or varied patterns of misspecification (Fig C.1 in S1 Appendix). To address these limitations, we propose a method called *BayesPowerNMF*. By leveraging a power posterior [12] and a sparsity-inducing prior [20], BayesPowerNMF provides nonparametric robustness to model misspecification and automated, principled selection of the number of latent processes. Through simulation studies and a comparison to existing methods on whole-genome data for six cancer types [25], we show that BayesPowerNMF finds more true processes and fewer spurious processes than competitors; see Fig 1 for two motivating illustrations.

The paper is organized as follows. In Methods, we introduce our proposed methodology. We present simulation results comparing our method to leading existing methods in Simulation Results. Then, in Application Results, we compare the methods in an application to whole-genome data from the Pan-Cancer Analysis of Whole Genomes Consortium (PCAWG). We conclude with a Discussion, including limitations and directions for future work.

## Methods

In this section, we describe the standard Poisson NMF model, our sparsity-inducing prior, the power posterior, our technique for choosing the power, and the overall workflow of our BayesPowerNMF method. NMF is based on the assumption that the data matrix $X=(X_{ji})\in {\mathbb{R}}_{+}^{J\times I}$ can be approximated by a low-rank factorization of the form


$$\begin{array}{c} X\approx LR, \end{array}$$

where the (unknown) matrices $L=(L_{jk})\in {\mathbb{R}}_{+}^{J\times K}$ and $R=(R_{ki})\in {\mathbb{R}}_{+}^{K\times I}$ have non-negative entries [26,27]. Here, ${\mathbb{R}}_{+}=\left[0,\infty \right)$ and we use bold font to indicate matrices. More formally, NMF solves


$$\begin{array}{c} \underset{L,R}{\text{arg,min}}\left\|LR-X\right\|\;\text{subject to}\;L\in {\mathbb{R}}_{+}^{J\times K}\text{ and }R\in {\mathbb{R}}_{+}^{K\times I}, \end{array}$$

where ‖·‖ is some appropriate choice of norm or divergence [28]. Since arbitrary multiplicative constants can be moved between ***L*** and ***R*** without affecting the product LR, we constrain the rows of ***R*** to sum to 1, that is, $\sum_{i=1}^{I}R_{ki}=1$ for all k=1,…,K. Low-rank factorizations such as this often provide valuable insights into the latent structure giving rise to the data.

In mutational signatures analysis, the rows of ***X*** correspond to samples j=1,…,J, the columns of ***X*** correspond to a pre-defined set of *I* non-overlapping mutation types, and each entry $X_{ji}\in \left\{0,1,2,\ldots \right\}$ represents the number of times that mutation type *m* is observed in sample *n*. The idea of the NMF model is that mutations arise due to multiple unknown mutational processes, and each such process generates mutations according to a distinct profile of frequencies. The *k*th row of ***R***, say $R_{k}=\left(R_{k1},\ldots ,R_{kI}\right)$, represents the frequencies with which mutation types i=1,…,I occur under the *k*th mutational process, and *R*_*k*_ is referred to as the *mutational signature* of this process. Thus, *K* represents the number of mutational processes represented in the model (that is, the number of latent factors). The loading *L*_*jk*_ represents the activity of process *k* in sample *j*.

### Model and sparsity-inducing prior

Our methodology starts with the *Poisson NMF model*, which for i=1,…,I,j=1,…,J, is given by $X_{ji}\sim \text{Poisson}\left(\sum_{k=1}^{K}L_{jk}R_{ki}\right)$ independently; or, in more compact matrix notation,


$$\begin{array}{c} X\sim \text{Poisson}\left(LR\right). \end{array}$$ (1)

This model is standard in mutational signatures analysis [17,29] and can be justified from first principles [20]. The parameters of the model are (***L***, ***R***) and the likelihood is


$$\begin{array}{c} \text{Poisson}\left(X|LR\right)=\prod_{j=1}^{J}\prod_{i=1}^{I}\text{Poisson}(X_{ji}\;|\sum_{k=1}^{K}L_{jk}R_{ki}). \end{array}$$ (2)

For the prior on ***R***, we use independent *M*-dimensional Dirichlet priors on the rows,


$$\begin{array}{c} R_{k}\sim {\text{Dirichlet}}_{I}\left(\alpha_{0},\ldots ,\alpha_{0}\right),\;k=1,\ldots ,K, \end{array}$$ (3)

where $\alpha_{0}>0$. For the prior on ***L***, since the number of active mutational processes is not known *a priori*, we employ the sparsity-inducing prior introduced by Zito and Miller [20]. Specifically, we place independent gamma priors on the loadings,


$$\begin{array}{c} L_{jk}\sim \text{Gamma}\left(a,\;a/\mu_{k}\right),\;j=1,\ldots ,J,\;k=1,\ldots ,K, \end{array}$$ (4)

where *a* > 0 is the shape and $a/\mu_{k}$ is the rate, so that $𝔼L_{jk}=\mu_{k}$ and hence $\mu_{k}$ is the mean prior loading for the *k*th mutational process. Finally, we give $\mu_{k}$ an inverse gamma hyperprior,


$$\begin{array}{c} \mu_{k}\sim \text{InvGamma}\left(a_{0},b_{0}\right),\;k=1,\ldots ,K, \end{array}$$ (5)

independently, where $a_{0}=J_{0}a+1$ and $b_{0}=\varepsilon \left(a_{0}-1\right)$ for some ε>0, a small nonzero constant. With these choices, the prior mean of $\mu_{k}$ becomes $𝔼\mu_{k}=\varepsilon$ and its full conditional mean is


$$\begin{array}{c} 𝔼\left[\mu_{k}|L,R,X,\mu_{1},\ldots ,\mu_{k-1},\mu_{k+1},\ldots ,\mu_{K})\right]=\frac{J_{0}}{J_{0}+J}\varepsilon +\frac{J}{J_{0}+J}\left(\frac{1}{J}\sum_{j=1}^{J}L_{jk}\right). \end{array}$$

This choice of hyperprior has a compressive property that induces column-wise sparsity in the loadings matrix under the posterior distribution. More precisely, given the data ***X***, it shrinks the $\mu_{k}$ value for inactive processes down to $\mu_{k}\approx \varepsilon$, while having a moderate shrinkage effect on the $\mu_{k}$ value for active processes. Consequently, the loadings *L*_*jk*_ for inactive processes shrink to small values ≈ε, while the loadings for active processes are only slightly affected by the hyperprior [20].

Thus, this Bayesian formulation provides automatic selection of the number of active signatures. Specifically, our choice of hierarchical prior favors small values of $\mu_{k}$ (near ε), which in turn favors small values of loadings. Consequently, any extra unneeded signatures will have their loadings shrunk to negligible values, effectively removing them from the model. In principle, the model can quantify uncertainty in the number of active signatures by sampling from the posterior and considering any $\mu_{k}$ values below a threshold, say 2ε, to be inactive. That said, the hyperprior we use is designed to concentrate on the smallest number of signatures that can fit the data sufficiently well. Consequently, the number of active signatures tends to exhibit little variability across posterior samples. See Zito and Miller [20] for details.

In our experiments, we set $\alpha_{0}=0.5$, *a* = 0.5, ε=0.001, and *J*_0_ = 10. Choosing $\alpha_{0}=0.5$ and *a* = 0.5 makes the prior weakly informative. Specifically, the prior on *R*_*k*_ is the Jeffreys prior for a multinomial likelihood and the conditional prior variance of *L*_*jk*_ is $Var\left(L_{nk}|\mu_{k}\right)=2\mu_{k}^{2}$. The value of ε does not matter much as long as it is significantly smaller than the smallest true mean loading. We find the choice of *J*_0_ = 10 to work well empirically for the size of data sets we consider (the largest being *J* = 326), providing sparsity without shrinking the $\mu_{k}$ values too strongly. Zito and Miller [20] considered the effect of *J*_0_ on the posterior behavior of the model, establishing theoretical and empirical results comparing using a fixed value such as *J*_0_ = 10 versus a growing value such as *J*_0_ = *J*. The former yields a traditional fixed-strength prior whereas the latter adaptively increases the penalization to match the strength of the data, leading to sparsity asymptotically as J→∞. Thus, a larger value of *J*_0_ may be appropriate for larger data sets.

### The power posterior

The standard Poisson NMF model works well on data generated from the model itself, but its performance suffers when the model is not exactly correct, as we demonstrate in Simulation Results. To address this issue, we employ the power posterior technique for improving robustness to misspecification [12].

Letting $\pi_{0}\left(\theta \right)$ denote the prior density on θ=(L,R,μ), p(X∣θ)=Poisson(X∣LR) the likelihood, and $Z\left(X\right)=\int p\left(X|\theta \right)\;\pi_{0}\left(\theta \right)\;d\theta$ the marginal likelihood, the standard posterior is


$$\begin{array}{c} \pi \left(\theta |X\right)=\frac{p\left(X|\theta \right)\;\pi_{0}\left(\theta \right)}{Z\left(X\right)}. \end{array}$$ (6)

For ξ∈[0,1], the *power posterior* is


$$\begin{array}{c} \pi_{\xi }\left(\theta |X\right)=\frac{p{\left(X|\theta \right)}^{\xi }\;\pi_{0}\left(\theta \right)}{Z_{\xi }\left(X\right)}, \end{array}$$ (7)

where $Z_{\xi }\left(X\right)=\int p{\left(X|\theta \right)}^{\xi }\;\pi_{0}\left(\theta \right)\;d\theta$ is the normalization constant. In particular, when ξ=0 or ξ=1, we recover, respectively, the prior or the standard posterior; that is, $\pi_{\xi =0}\left(\theta |X\right)=\pi_{0}\left(\theta \right)$ and $\pi_{\xi =1}\left(\theta |X\right)=\pi \left(\theta |X\right)$. As shown by Miller and Dunson [12], using a power posterior with 0<ξ<1 provides nonparametric robustness for misspecified latent variable models, particularly when the latent dimensionality is unknown, such as in mutational signatures analysis. Furthermore, Medina et al. [30] showed that—compared to using the standard posterior—using the power posterior of a misspecified model is closer to the standard posterior of a more flexible well-specified model, in terms of Kullback–Leibler divergence.

To perform inference, we use the Stan probabilistic programming system [31] to draw Markov chain Monte Carlo (MCMC) samples from the power posterior of the NMF model. Specifically, the log target density is


$$\begin{array}{c} \log\pi_{\xi }\left(\theta |X\right)=const+\log\pi_{0}\left(\theta \right)+\xi \sum_{j=1}^{J}\sum_{i=1}^{I}\log\text{Poisson}(X_{ji}\;|\;\sum_{k=1}^{K}L_{jk}R_{ki}). \end{array}$$ (8)

Using Stan makes it a simple matter of multiplying the log-likelihood part of this function by ξ to target the power posterior rather than the standard posterior. For the MCMC sampler settings, we use four chains, each with 10,000 samples after a burn-in of 10,000 iterations. We choose the chain with the largest approximate marginal likelihood, using the approximation of Pritchard et al. [32].

### Choosing the power

The power ξ should be selected to reflect the severity of the model misspecification [12,30]. When there is no misspecification—that is, when the data are generated by the assumed model—we would ideally take ξ=1, which corresponds to the standard Bayesian posterior. When there is misspecification, smaller values of ξ are preferable. However, it can be difficult to choose ξ in a data-driven way.

We propose a simulation-driven approach to choosing ξ. First, select a set of *pilot signatures* and estimate loadings to fit the input data using these pilot signatures. Next, generate pilot data from several plausible data generating processes (DGPs) based on the pilot signatures and corresponding estimated loadings. Then, fit each pilot data set using a range of ξ values, and select a value of ξ that performs well across all of the pilot data sets. For mutational signatures discovery, we use the COSMIC v2 signatures [7,33] as the pilot signatures since they are well separated, which facilitates interpretation of the results for power selection. Meanwhile, we use COSMIC v3 signatures for benchmarking and interpretation on real data.

By exploring simulated data from DGPs that are not equal to the assumed model, but are anchored at parameters fit to the input data, we are able to select a ξ that yields robustness to plausible perturbations within an appropriate data-driven neighborhood of the model. This yields robustness to any DGPs that would require a similar power ξ, not just to the specific forms of DGP used to generate pilot data. Thus, it is not critical to get the DGPs exactly right—just to make them sufficiently rich and plausible.

### Simulating data generating processes

Given a matrix of pilot signatures $R^{⋆}$ and corresponding matrix of loadings $L^{⋆}$, we generate pilot data sets using the following four DGPs based on $R^{⋆}$ and $L^{⋆}$:

1. The well-specified DGP generates data from the assumed Poisson NMF model, as in Equation (1):


$$\begin{array}{c} X\sim \text{Poisson}\left(L^{⋆}R^{⋆}\right). \end{array}$$

2. The α-contaminated DGP generates data from the Poisson NMF model, but with an additional, subject-specific “contamination signature” ${\tilde{R}}_{n}$: for α∈(0,1),


$$\begin{array}{c} {\tilde{R}}_{j}\sim {\text{Dirichlet}}_{I}\left(1,\ldots ,1\right),\;n=1,\ldots ,N,\\ X\sim \text{Poisson}\left(\left(1-\alpha \right)L^{⋆}R^{⋆}+\alpha (L^{⋆}\;1_{K\times I})⊙\tilde{R}\right), \end{array}$$ (9)

where $1_{K\times I}$ denotes the *K* × *I* matrix of ones, ⊙ denotes entry-wise multiplication, and $\tilde{R}$ is the matrix with rows ${\tilde{R}}_{1},\ldots ,{\tilde{R}}_{J}$. The contamination signature ${\tilde{R}}_{n}$ is different for each sample and is intended to model a low level of idiosyncratic mutational noise unique to each sample that is unlikely to represent a biologically meaningful mutational process. Equation 9 gives ${\tilde{R}}_{n}$ a loading of $\alpha \sum_{k=1}^{K}L_{nk}^{⋆}$, and the existing loadings $L_{jk}^{⋆}$ are downweighted by a factor of 1−α to keep the total loading invariant.

3. The γ-perturbed DGP generates each sample using slightly perturbed versions of each signature, with the perturbations being subject-specific: given γ>0 and $\beta_{1},\ldots ,\beta_{K}>0$,


$$\begin{array}{c} R_{k}^{\left(j\right)}\sim {\text{Dirichlet}}_{I}\left(\beta_{k}R_{k1}^{⋆},\ldots ,\beta_{k}R_{kI}^{⋆}\right),\;\;n=1,\ldots ,J,\;k=1,\ldots ,K,\\ X_{j}\sim \text{Poisson}\left(L_{n}^{⋆}R^{\left(j\right)}\right),\;\;j=1,\ldots ,J, \end{array}$$

where $L_{n}^{⋆}$ is the *n*th row of $L^{⋆}$, $R^{\left(j\right)}$ is the matrix with rows $R_{1}^{\left(j\right)},\ldots ,R_{K}^{\left(j\right)}$, and *X*_*j*_ is the *n*th row of ***X***. The constants $\beta_{k}$ control the size of perturbations, and are set such that the expected *cosine error* between $R_{k}^{\left(j\right)}$ and $R_{k}^{⋆}$ is approximately γ, where the cosine error is defined as $1-R_{k}^{⊤}R_{\ell }^{⋆}/\left(\|R_{k}\|\|R_{\ell }^{⋆}\|\right)$; see Appendix A in S1 Appendix for details. This DGP reflects the possibility that each mutational process might behave slightly differently across tumors, for instance due to the tumor microenvironment or primary tumor tissue [34], interactions of DNA damage and repair mechanisms [35,36], different driver mutations within a DNA repair mechanism [37], or differing opportunity counts [4,17].

4. The κ-overdispersed DGP generates data with higher variance than the well-specified data, to reflect the fact that there is often additional variability due to subject- and mutation-type specific effects that may be biological or technical: given κ>1,


$$\begin{array}{c} X\sim \text{NegBin}\left(L^{⋆}R^{⋆},\kappa \right), \end{array}$$

where NegBin(μ,κ) denotes the negative binomial distribution with mean μ and variance κμ.

In our experiments, we use α=0.02, γ=0.0025, and κ=2 for the settings of these DGPs. These settings are chosen to reflect plausible levels of biological and technical perturbations from the Poisson model. Specifically, α=0.02 equates to 2% of observed mutations originated from some source of contamination and γ=0.0025 is a threshold where the simulated signatures are almost always still recognizable as coming from the original reference signature (99% of simulated signatures have cosine error < 0.1). We set κ=2 to reflect the empirical overdispersion seen in loadings through exploratory analysis of real mutation counts data from the PCAWG project.

### Complete workflow

**Fig 2:**
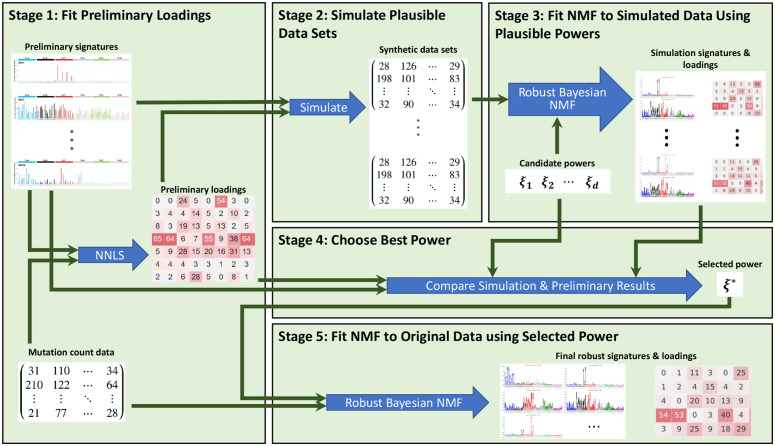
BayesPowerNMF workflow for robust mutational signature discovery. *(1)* Pilot signatures from a reference are used to estimate loadings to fit the input data. *(2)* These signatures and loadings are used to simulate pilot data sets from DGPs representing plausible perturbations of the model. *(3)* The power posterior for the NMF model is applied to each combination of candidate power $\xi_{i}$ and simulated data set ***X***_*j*_. *(4)* We identify the power that best recovers the pilot signatures and loadings across all of the pilot data sets. *(5)* Using the selected power, we apply the power posterior for the Bayesian NMF model to the original input data.

In detail, the overall workflow of our proposed method, *BayesPowerNMF*, consists of the following five stages (see Fig 2):

(1) *Define pilot signatures and loadings.* To tailor the pilot data sets to the input data set, we define the pilot signatures $R^{⋆}$ and loadings $L^{⋆}$ as follows. The exact approach to generate such pilot datasets will be problem dependent. In the mutational signatures context, we use non-negative least squares [38] to estimate loadings for the 30 COSMIC v2 reference signatures [7,33] for each individual in the cohort. We then drop any reference signatures that, based on the estimated loadings, do not contribute at least 2% of the total mutation count in the cohort or at least 10% of the mutation count of any individual, so that each remaining signature is well represented. To maintain the accuracy of the loadings, the remaining loadings are not re-fit.
(2) *Generate pilot data sets.* For each of *T* DGPs, generate a pilot data set based on parameters $L^{⋆}$ and $R^{⋆}$, which results in *T* simulated data sets $X_{1},\ldots ,X_{T}$. As in the first stage, the exact choice of *T* and the process of generating the simulated data sets is problem-specific. We use *T* = 4: the well-specified DGP plus the three misspecified DGPs described in Simulating data generating processes, with parameters α=0.02, γ=0.0025, and κ=2. The rationale for these parameter choices is provided in Simulating data generating processes.
(3) *Estimate parameters for a range of* ξ *values.* For each power ξ in a range of candidate values, for each pilot data set, we estimate ***L*** and ***R*** using the mean of the power posterior (The power posterior) and match the estimated signatures *R*_*k*_ to the pilot signatures $R_{k}^{⋆}$. Matching *R*_*k*_ to $R_{k}^{⋆}$ is done using the Hungarian algorithm for optimal bipartite matching [39] with cosine error as the assignment cost.
(4) *Choose* ξ *to maximize accuracy across pilot data sets.* We choose the largest power ξ that accurately recovers $L^{⋆}$ and $R^{⋆}$ according to the following metrics (see Fig C.2 in S1 Appendix): *(i)* recovering as many true signatures as possible with small cosine error (with strict upper bound of 0.3), and *(ii)* not inferring any *spurious signatures* that fail to match any true signature.
(5) *Apply power posterior to the input data.* Using the selected power ξ, we apply the power posterior to the original input data ***X*** to estimate ***L*** and ***R***. This yields the final estimates of ***L*** and ***R*** produced by the workflow.

## Results

### Simulation results

In this section, we compare performance on synthetic data simulating six cancer types, using the four data generating processes described in Simulating data generating processes. We compare four NMF methods for inferring mutational signatures: (1) SigProfilerExtractor, a version of the algorithm used to define the COSMIC signatures [5,24], (2) SignatureAnalyzer, based on a Bayesian point estimation algorithm [18] used in several previous publications [16,40–42], (3) SigMoS, which uses an NMF algorithm based on a negative binomial likelihood and subject-specific overdispersion parameters to account for certain kinds of misspecification [22], and (4) BayesPowerNMF, our proposed method.

#### Simulated data.

We generate synthetic data sets in the well-specified case for six cancer types (lung adenocarcinoma, stomach, melanoma, ovary, combined breast, and liver) and in the three misspecified cases (α-contaminated, γ-perturbed, and κ-overdispersed) for three cancer types (lung adenocarcinoma, ovary, and liver). These simulated data sets are generated as in stages 1 and 2 of the BayesPowerNMF workflow with the input data set being a mutation count matrix for the corresponding cancer type from the PCAWG project [25]. To increase realism, we do not modify the number or loadings of the signatures used to generate the simulated data. Therefore, in some cases there are more signatures than any method could reasonably recover given the data set sizes. This choice reflects the reality of there being a “long tail” of uncommon or low-activity mutational processes that may be hard to discover. In addition, we consider randomly subsampled data sets of sizes 20, 30, 50, 80, 120, 170, and 230 from the simulated well-specified liver data set of 326 samples. Thus, altogether, starting from 6 real data sets (one for each cancer type), we generate 15 complete synthetic data sets (6 well-specified and 3 × 3 misspecified) plus 104 data sets subsampled from the complete well-specified liver data set (32 of size *N* = 20, 20 of size *N* = 30, 12 of size *N* = 50, and 10 each of the remaining sizes). Using each of these synthetic data sets as input, we then apply each of the four competing methods listed above: SigProfilerExtractor, SignatureAnalyzer, SigMoS, and BayesPowerNMF.

#### Performance evaluations.

We evaluate performance by measuring how well the ground-truth signatures and loadings are recovered in terms of several metrics. Here, ground truth is known since the data is simulated. First, we compute the optimal matching (in terms of cosine error) between estimated and true signatures using the Hungarian algorithm [39]. We then quantify how accurately each signature is recovered by computing the cosine error between each pair of matched signatures. We compute the precision and recall for recovering the true signatures by setting a threshold on cosine error and considering a true signature to be correctly recovered if the cosine error of its matching estimated signature is within this threshold. Precision is defined as (# correctly recovered) / (# estimated signatures) and recall is (# correctly recovered) / (# true signatures). Precision and recall curves are generated by varying the threshold. Additionally, to provide a comprehensive visual summary of the results, we use bubble plots to display all matched pairs of signatures, along with their cosine errors and loadings, for each method and each data set.

#### Simulation results.

**Fig 3:**
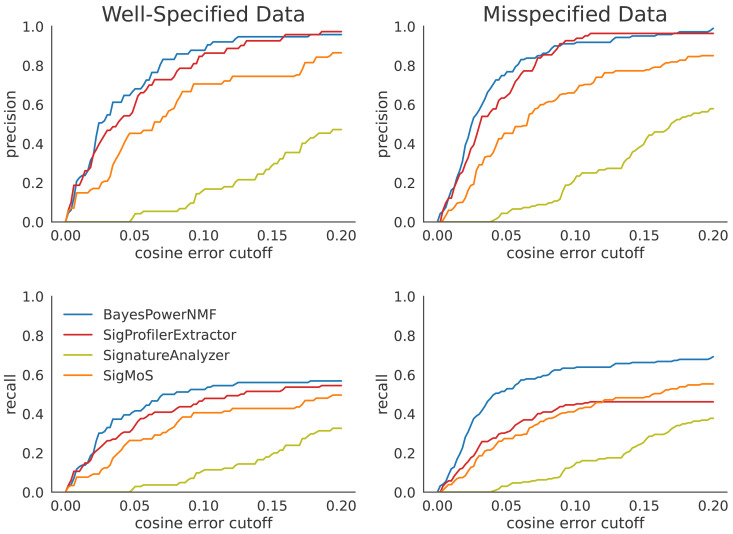
Precision and recall for well- and misspecified simulated mutation counts. *(Top)* Precision curves for each method as a function of cosine error threshold, averaged across the simulated well-specified data sets (left) and misspecified data sets (right). (*Bottom*) Same for the recall curves. BayesPowerNMF has the highest precision and recall overall. SigProfilerExtractor performs nearly as well as BayesPowerNMF in the well-specified settings, but its recall is lower under misspecification. SigMoS and especially SignatureAnalyzer exhibit lower precision and recall in these simulations.

**Overall precision and recall:** Fig 3 shows the precision and recall for each of the methods, over a range of cosine error thresholds from 0 to 0.2. These curves are averaged over all of the well-specified data sets (left side) and misspecified data sets (right side). BayesPowerNMF provides the highest precision and recall at nearly every threshold. In particular, it has much higher recall in the misspecified settings. SigProfilerExtractor performs only somewhat worse than BayesPowerNMF in the well-specified settings, but its recall suffers under misspecification. SigMoS performs much more similarly to SigProfilerExtractor under the misspecified data sets, which makes sense as SigMoS explicitly models some forms of misspecification.

The signatures missed by SigProfilerExtractor tend to be ones with smaller true loading; see Fig C.3 in S1 Appendix. One reason for this may be because SigProfilerExtractor employs a consensus bootstrap approach that requires agreement across the estimates based on different bootstrap data sets. To compare the performance for each signature individually, we plot the cosine error from the ground truth for SigProfilerExtractor versus BayesPowerNMF in Fig C.4 in S1 Appendix; this illustrates that BayesPowerNMF tends to exhibit better performance on a signature-by-signature basis.

We find that SignatureAnalyzer exhibits worse performance across all scenarios in terms of both precision and recall. The reason for this is that SignatureAnalyzer estimates many signatures, but the estimated signatures have a high cosine error relative to the matching true signatures. Although SignatureAnalyzer is based on a Bayesian model, it employs *maximum a posteriori* estimation rather than posterior uncertainty quantification, and does not benefit from the robustness of the power posterior. SigMoS performs better than SignatureAnalyzer but worse than SigProfilerExtractor.

**Fig 4:**
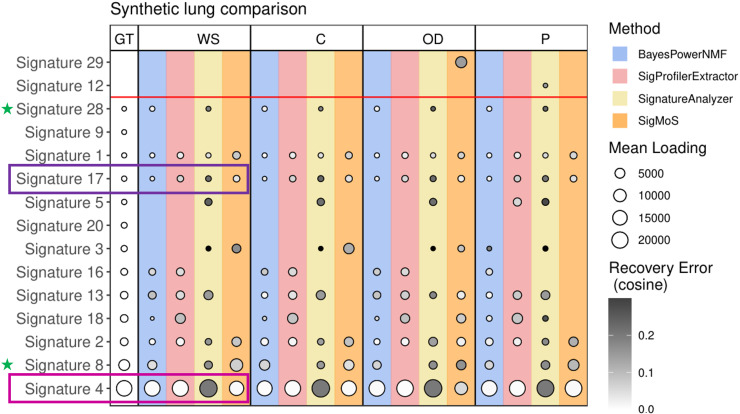
Comparison of results on simulated lung adenocarcinoma data sets, via a bubble plot showing the loading and cosine error of the estimated signatures. Rows represent the true COSMIC v2 signatures, ordered by the ground truth (GT) loading. Each column shows the results for a given method (blue = BayesPowerNMF, red = SigProfilerExtractor, yellow = SignatureAnalyzer, orange = SigMoS) on a given data set (WS = well-specified, C = α-contaminated, OD = κ-overdispersed, P = γ-perturbed). Presence of a bubble means an estimated signature matched that true signature. Bubble size = estimated loading, bubble shade = cosine error between estimated and true, and a bubble above the red line represents an estimated signature that did not match any true signature used to generate the data. Overall, BayesPowerNMF consistently finds the most signatures and has the lowest cosine errors.

**Lung adenocarcinoma results:** To understand what each method is doing at a more granular level, Fig 4 shows the cosine error and loading for each signature. The true COSMIC signatures are listed on the left, and each column summarizes the results from a given method on a given data set. The size of each bubble represents the loading given by that method to the estimated signature that was matched to the true signature listed on the left. The shade of the bubble represents the cosine error between the estimated signature and the matching true signature. The left-most column (labelled GT) shows the ground truth loadings used to generate the simulated data, and we order the rows (signatures) according to these ground truth loadings. Therefore, a method is performing well on a given data set if *(i)* it has bubbles for the same signatures as GT, *(ii)* the sizes of the bubbles are similar to GT, and *(iii)* the shade of the bubble is white or light gray. Methods with bubbles above the red line estimated a spurious signature that does not match to any of the true signatures used to generate the data.

For the simulated lung adenocarcinoma data sets, which have *N* = 38 samples, Fig 4 shows that BayesPowerNMF tends to recover more true signatures with lower cosine error than the other methods. SigProfilerExtractor performs second best, but misses some signatures (in particular, Signatures 8 and 28, indicated by green stars) and tends to have slightly higher cosine error. Note that Signature 8 was not recovered by SigProfilerExtractor in any of the cases, even though this signature has a large true loading (second largest out of all the signatures). SignatureAnalyzer does recover many signatures that match to the true signatures, but they have very high cosine error, even for signatures with high true loading such as Signature 4 (5B). On the γ-perturbed data, SignatureAnalyzer also produces a spurious signature that matches to COSMIC Signature 12, which was not used to generate the data. SigMoS produces the fewest signatures of the four methods in all but the κ-overdispersed data. Except for a spurious signature inferred from the κ-overdispersed data, signatures inferred by SigMoS have a comparable recovery error range to those inferred by SigProfilerExtractor.

**Fig 5:**
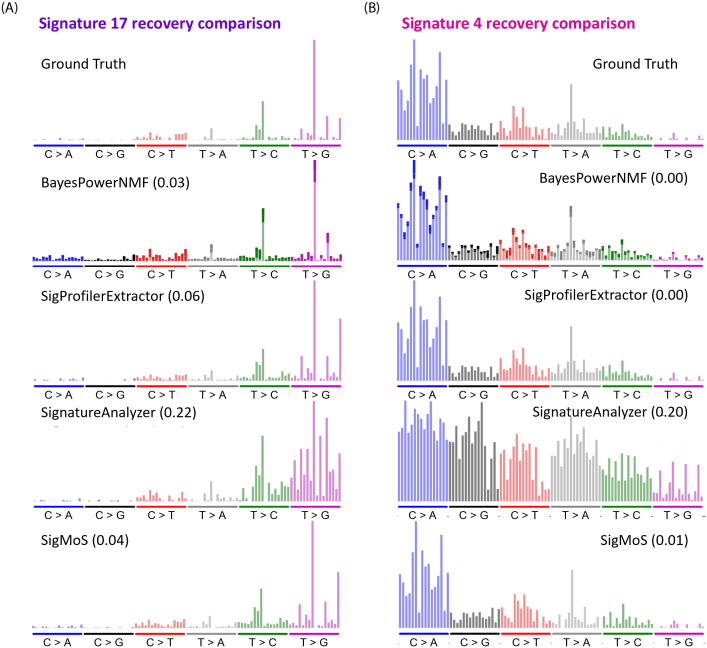
Comparison of estimated signatures on simulated lung adenocarcinoma data sets. ***(A)*** True Signature 17 and matching estimated signatures (along with their cosine error, in parentheses) for each method on the WS data. For BayesPowerNMF, the bolded section of each bar for represents 95% credible interval and the thin white line in the middle represents the posterior mean. ***(B)*** Same panel A but for Signature 4.

Fig 5A shows true Signature 17 and the matching estimated signatures for each method on the well-specified (WS) synthetic lung adenocarcinoma data (corresponding to purple box in Fig 4). This is an example of a signature with relatively small true mean loading and, in comparison with Signature 4, it also has many more near-zero values in the signature itself. BayesPowerNMF recovers the signature with the lowest cosine error (0.03), followed by SigMoS (0.04), then SigProfilerExtractor (0.06), and finally SignatureAnalyzer (0.22). BayesPowerNMF also provides uncertainty quantification in the signature vector, indicated in the plot by the darker region at the top of each bar, which represents a 95% credible interval.

Similarly, Fig 5B shows true Signature 4 and the matching estimated signatures on the well-specified synthetic lung adenocarcinoma data (corresponding to magenta box in Fig 4). This is an example of a signature with large true mean loading (the largest in this simulated data). All four methods estimate a signature that matches to Signature 4, with estimated loading similar to the true loading. Furthermore, BayesPowerNMF, SigProfilerExtractor, and SigMoS recover the true signature with very low cosine error (<0.01). However, SignatureAnalyzer’s matching signature has a high cosine error (0.20). For BayesPowerNMF, the 95% credible regions (bolded regions of the bars) tend to indicate less uncertainty in the estimates for Signature 4 compared to 17, especially in the mutation types with smaller rates; this makes sense since Signature 4 has higher true mean loading and thus there is more information about it in the data.

**Fig 6:**
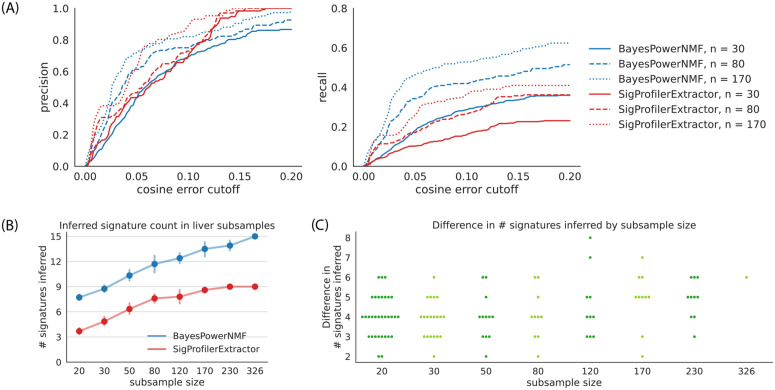
Results for subsampled liver cancer data sets of increasing size. ***(A)*** Precision and recall of matched signatures for a selection of subsample sizes, as a function of the cosine error threshold for defining a correct match. BayesPowerNMF almost always strictly dominates SigProfilerExtractor in recall at all sample sizes, and has comparable or better precision for cosine error thresholds values of practical utility (< 0.1). ***(B)*** Number of signatures estimated (with whiskers representing 95% confidence intervals) at each sample size. BayesPowerNMF recovers more signatures than SigProfilerExtractor at all sample sizes. The true number of signatures is 21. ***(C)*** Difference between number of signatures estimated by BayesPowerNMF and SigProfilerExtractor for each subsampled synthetic liver data set. Each dot represents one subsampled data set.

**Subsampled liver cancer data sets of varying size:** Next, we evaluate the effects of sample size and sampling variability by considering the subsampled data sets of varying size taken from the complete simulated well-specified liver cancer data set of *N* = 326 samples. The full cohort of *N* = 326 samples was generated using 21 ground truth signatures. From this, we repeatedly subsampled data sets of various sizes and ran each method on each subsampled data set. Fig 6 summarizes the results for BayesPowerNMF and SigProfilerExtractor on these data; SignatureAnalyzer and SigMoS are excluded from this analyses due to overall poor performance. Fig 6A plots the precision and recall curves as a function of cosine error threshold, as in Fig 3. As before, we see that BayesPowerNMF outperforms SigProfilerExtractor, particularly in terms of recall. SigProfilerExtractor does exhibit slightly higher precision at higher cosine error thresholds, but this comes at the expense of much lower recall. Fig 6B shows the number of signatures inferred by each method as a function of the size of the data set. This shows that BayesPowerNMF requires a much smaller sample size to recover the same number of signatures as SigProfilerExtractor. For instance, BayesPowerNMF recovers an average of 9 signatures on a data set of size *N* = 30, whereas SigProfilerExtractor requires *N* = 230 to recover this many signatures on average. Thus, BayesPowerNMF accurately recovers the same number of signatures with a fraction of the sample size.

To examine the effects of sampling variability, Fig 6C shows the difference in the number of signatures inferred by BayesPowerNMF and SigProfilerExtractor for each individual subsampled data set. On every individual data set, BayesPowerNMF always recovers at least 2 more signatures than SigProfilerExtractor, and sometimes as many as 7 or 8 more.

#### Summary of simulation results.

Our simulation results demonstrate that BayesPowerNMF has improved performance compared to leading methods—enabling the discovery of a greater number of meaningful signatures—while also being robust to plausible model misspecification. One downside of BayesPowerNMF is that the additional robustness obtained in misspecified settings comes at a cost of reduced performance in well-specified settings compared to using the standard Bayesian posterior; see Fig C.6 in S1 Appendix. Overall, we find that BayesPowerNMF has the following specific advantages.

**Higher precision:** Compared to SigProfilerExtractor, SignatureAnalyzer, and SigMoS, BayesPowerNMF tends to have higher precision. This holds across different DGPs (Fig 3 and Fig C.4 in S1 Appendix) and across sample sizes (Fig 6A), especially for smaller cosine error cutoffs that would produce recognizable signature matches (such as 0.05 or 0.1).

**Higher recall:** BayesPowerNMF consistently outperforms the alternatives in terms of recall, particularly in misspecified cases (Fig 3 and 6A and Table C.1 in S1 Appendix). In the misspecified cases, SigProfilerExtractor tends to be conservative, inferring too few signatures (Fig 3 and Table C.2 in S1 Appendix). SignatureAnalyzer and SigMoS sometimes infer more signatures, but many of the estimated signatures either have very large cosine error (> 0.2) or do not even get matched to one of the true signatures used to generate the data.

**Uncertainty quantification:** As a fully Bayesian method, BayesPowerNMF quantifies uncertainty in the signatures as well as the loadings via posterior samples produced by MCMC (Fig 5). We find that the model’s reported uncertainty in the signatures is correlated with the actual recovery error (Fig C.5 in S1 Appendix), indicating that the uncertainty quantification is meaningful. In contrast, SigProfilerExtractor, SignatureAnalyzer, and SigMoS do not provide uncertainty quantification in either the signatures or the loadings.

**Few spurious signatures:** We consider an estimated signature to be “spurious” if it is matched to a COSMIC signature that was not used to generate the simulated data set. Out of the 15 complete simulated data sets we consider, BayesPowerNMF infers a spurious signature in just one data set (see Table C.1 and C.2 in S1 Appendix). SignatureAnalyzer inferred spurious signatures in several synthetic data sets, even in the well-specified case (see Table C.1 in S1 Appendix).

### Application results

We compare the results of BayesPowerNMF and SigProfilerExtractor on whole-genome sequencing (WGS) data from the PCAWG project [25]. SignatureAnalyzer and SigMoS are excluded from this comparison due to overall poor performance. We consider the PCAWG WGS data for the same six cancer types as in the simulation study: lung adenocarcinoma (*N* = 38), stomach (*N* = 75), melanoma (*N* = 107), ovary (*N* = 113), combined breast cancers (*N* = 214), and liver (*N* = 326). While any set of mutation types could be used to define the input count data matrices, we consider the 96 types of single-base substitutions (SBSs) since these are the most commonly used set for mutational signatures analyses. We run BayesPowerNMF and SigProfilerExtractor on the *N* × 96 count data matrix for each cancer type separately.

To evaluate performance, we use the COSMIC v3 signatures as a proxy for the true signatures. (Note, however, that we still use COSMIC v2 in the BayesPowerNMF workflow when defining pilot signatures for calibration.) The COSMIC signature database is a curated reference set of mutational signatures that is commonly used in cancer genomics studies. Version 3 of the COSMIC signatures are based on 2,780 cancer genomes from the PCAWG project—along with a number of other available cancer genomes—spanning a wide range of cancer types [8,25]. The COSMIC v3 signatures were defined by running a version of SigProfilerExtractor on each cancer type separately, and combining the results to construct a set of consensus signatures. Thus, one would expect SigProfilerExtractor to perform very well in these comparisons, since it was instrumental in defining our proxy for ground truth.

Since the actual ground truth is unknown on real data, we quantify method accuracy using using three imperfect proxies for quality. However, it is also important that the inferred signatures correspond to true signatures and are accurately estimated; in other words, it is important to have high precision. Thus, second, we compute the error with which the COSMIC signatures are reconstructed. More precisely, we assign the inferred signatures to the best-matching COSMIC v3 signatures using the Hungarian algorithm, and compute the cosine error between each matched pair. It is desirable to have smaller error, since this indicates greater accuracy in recovering the current best understanding of ground truth. Third, we compare the estimated loadings for each method to the loadings presented by the PCAWG consortium, which they obtained by running a variant of non-negative least squares (NNLS) using the complete set of COSMIC signatures, rather than using NMF on a cancer-by-cancer basis [8]. We refer to these as the “COSMIC+NNLS” loadings.

Table 1 and Fig C.7 in S1 Appendix summarize the results.

**Table 1:** Number of signatures inferred in PCAWG mutation counts data. Number of signatures inferred by each method in PCAWG mutation counts data for six cancer types (Application Results), treating COSMIC+NNLS as ground truth. (SPE = SigProfilerExtractor; BPN = BayesPowerNMF; COSMIC = COSMIC+NNLS.).

	Lung			Stomach			Skin			Ovary			Breast			Liver		
BPN	10		*+1*	9	+1	*+5*	5	+1		8	+1	*+3*	6		*+2*	12	+4	*+9*
SPE	7		*+1*	7	+3	*+4*	5	+1	*+4*	6	+1	*+1*	8	+2		7	+4	*+3*
COSMIC	12			15			12			11			12			21		

In each entry of the form X + Y *+ Z* in the table, X = the number of estimated signatures that were matched to a COSMIC v3 signature associated with the cancer type in [8] with a cosine error of <0.2, Y = the number of estimated signatures that were matched to a COSMIC v3 signature associated with the cancer type in [8] with a cosine error of ≥0.2, and *Z* = the number of estimated signatures that were matched to a COSMIC v3 signature that was *not* associated with the cancer type in [8].

#### Number of signatures inferred.

In four of the six cancer types, BayesPowerNMF estimates more signatures than SigProfilerExtractor (Table 1). Furthermore, in one of the two cancer types where SigProfilerExtractor infers more signatures (melanoma), some of the estimated signatures appear to be near duplicates rather than authentically different signatures (Fig C.8 in S1 Appendix). In many cases, BayesPowerNMF recovers almost as many signatures as COSMIC+NNLS (1 and Fig C.7 in S1 Appendix), even though BayesPowerNMF is only using a small subset of data from one cancer type whereas COSMIC+NNLS is effectively using ≈3,000 samples.

#### Reconstruction error.

Both methods perform roughly equally well in the accuracy with which they reconstruct COSMIC signatures. Indeed, the difference between the average cosine errors for BayesPowerNMF and SigProfilerExtractor inferred signatures (0.146 and 0.133, respectively) is not statistically significant (*p* = 0.9265, Mann–Whitney rank test). This is despite the fact that (1) SigProfilerExtractor is at an advantage since the COSMIC signatures were constructed using its algorithm and (2) BayesPowerNMF recovers a greater proportion of signatures (Table 1).

#### Loadings.

In most cases, the loadings estimated by BayesPowerNMF and SigProfilerExtractor are of similar magnitude to one another and to the COSMIC+NNLS loadings, at least for the signatures that they have in common (Fig C.7 in S1 Appendix). However, on the melanoma data, SigProfilerExtractor gives large loadings to two apparently spurious signatures that have high cosine error with their nearest COSMIC v3 matches (SBS30 and SBS11).

Overall, the real data results lead to similar conclusions as the simulation study. These findings indicate that our proposed method provides improved accuracy and robustness to model misspecification compared to the state-of-the-art, both on synthetic examples and real biological data. It is notable that SigProfilerExtractor appears to perform more poorly on the real PCAWG data than on our simulated misspecified counts data, compared to BayesPowerNMF. This suggests that the modes of misspecification in real mutation count data are more complex than is captured in each of our DGPs for misspecified counts, but this is accounted for in BayesPowerNMF due to the flexibility of the power posterior.

Finally, these results suggest that the precise choice of pilot signatures does not strongly affect the final results from BayesPowerNMF. The BayesPowerNMF workflow uses COSMIC v2 for the pilot signatures, whereas we ultimately compare to COSMIC v3. Notably, BayesPowerNMF successfully infers v3 signatures on this data, including ones with no corresponding signature in v2 (specifically, SBS31 and above in Fig C.7 in S1 Appendix).

## Discussion

Our results elucidate specific shortcomings of two of the standard methods for mutational signature discovery, SigProfilerExtractor and SignatureAnalyzer. SigProfilerExtractor uses a consensus bootstrap approach that resamples the mutation counts, which may explain why it tends to miss more signatures with small loadings (Fig C.3 in S1 Appendix). BayesPowerNMF does not have this issue since it uses all of the available data. This suggests that the SigProfilerExtractor results might be improved by using a version of bootstrap with continuous weights, such as the Bayesian bootstrap, rather than multinomial bootstrap.

Furthermore, while SigProfilerExtractor is generally more conservative in the sense that it recovers fewer signatures than BayesPowerNMF, it can still overfit the data in cancer types with high mutational burden (such as melanoma; see Fig C.8 in S1 Appendix) or many samples. This makes sense, since these are the cases in which we would expect the negative effects of misspecification to be most pronounced [12,13]. BayesPowerNMF avoids this issue by employing a power posterior to improve robustness to misspecification.

Similarly, a disadvantage of SignatureAnalyzer is that it relies heavily on the correctness of the Poisson NMF model [20]. While SigProfiler’s use of bootstrapping can somewhat mitigate the effects of misspecification [43,44] and BayesPowerNMF’s use of the power posterior provides robustness, SignatureAnalyzer has no mechanism to compensate for model misspecification. As a result, it appears that SignatureAnalyzer is particularly negatively affected by misspecification. Furthermore, SignatureAnalyzer uses an estimation algorithm based on several heuristic approximations to the objective function [18]. The use of these heuristics may explain why SignatureAnalyzer was not able to accurately recover the true signatures even on the simulated well-specified data sets, a setting in which we would expect a model-based method to perform well. In contrast, BayesPowerNMF does not suffer from this issue, since it does not employ any approximations other than MCMC sampling. So, as expected, it exhibits good performance on the well-specified data.

We also consider the method SigMoS, which explicitly accounts for some model misspecification by fitting a negative binomial NMF model with a subject-specific overdispersion parameter. While it largely performed similarly to SigProfilerExtractor with somewhat higher recovery error, SigMoS demonstrated significant overfitting for larger cohorts, such as for liver (Table C.2 in S1 Appendix). We find that the rigid imposition of an alternate NMF model is not sufficient to account for the varied modes of model misspecification that may arise in mutational signatures inference and other NMF settings, which the flexible power posterior approach in BayesPowerNMF is able to address.

The main disadvantage of BayesPowerNMF is that, when using MCMC techniques, it becomes computationally prohibitive for larger data sets. However, recent advances in variational inference methods for Bayesian NMF models, including applications to mutational signatures, could be integrated into the workflow with minimal modifications [45,46]. We leave a thorough investigation of this direction for future work. A related disadvantage is the somewhat complicated workflow for selecting the power ξ to be used in the power posterior. It would be preferable to have a simpler method of determining an appropriate power for a given set of plausible data generating processes.

Overall, BayesPowerNMF yields superior performance by *(i)* using all the available data and employing a full Bayesian model to extract as much information as possible from the data, while *(ii)* using a power posterior to obtain robustness to model misspecification without assuming a particular form of misspecification.

## Supporting information

S1 Appendix This supporting document contains all supplementary discussion, tables, and figures cited in the main text. (PDF)
