# Supplementary material for: Robust discovery of mutational signatures using power posteriors
Source: PLoS Comput Biol. 2026 Jun 11;22(6):e1014372. doi: 10.1371/journal.pcbi.1014372 (PMC13274926; doi:10.1371/journal.pcbi.1014372)
Supplement: S1 Appendix — (PDF) [file pcbi.1014372.s001.pdf]

Supplementary Methods and Results for "Robust discovery of mutational signatures using power posteriors"

Catherine Xue[1], Jeffrey W. Miller[1], Scott L. Carter[2], Jonathan H. Huggins* [3,4]
1 Department of Biostatistics, Harvard University, Boston, Massachusetts, USA

2 Department of Data Science, Dana–Farber Cancer Institute, Boston, Massachusetts, USA
3 Department of Mathematics & Statistics, Boston University, Boston, Massachusetts, USA
4 Faculty of Computing & Data Science, Boston University, Boston, Massachusetts, USA

A Details on perturbed data-generation process

Naïvely, one might try to simulate sets of perturbed mutational signatures by sampling from Dirichlet $(\beta R_k^\star)$ using the same concentration parameter $\beta > 0$ for all pilot signatures $R_k^\star$. However, in terms of cosine error, this leads to very different magnitudes of perturbation depending on the sparsity or flatness of $R_k^\star$ [1]; see Fig A.1.

We quantify the "flatness" of a signature $R_k^\star$ via

$$\text{flatness}\left(R_k^\star\right) = \frac{1}{96\left\|R_k^\star\right\|^2}. \tag{A.1}$$

Larger values indicate a flatter signature and smaller values indicate a spikier signature. This expression is based on the well-known effective sample size formula used in importance sampling. Through simulations using each $R_k^\star$ in the set of COSMIC v2 signatures, we observe that for a range of concentration parameters β ranging from 500 to 10,000, the mean cosine error for Dirichlet $(\beta R_k^\star)$ has a very strong positive linear correlation with flatness $(R_k^\star)$ (Fig A.2A). Further, the slopes of the best-fit line obtained with ordinary least squares (OLS) linear regression are strongly linearly correlated with β on a log-log scale (Fig A.2B).

Based on these observations, we use OLS linear regression to fit the empirical relationship between flatness $(R_k^\star)$, the desired mean cosine error $\gamma \in (0, 1)$, and the concentration parameter β:

$$\beta\left(R_k^\star\right) = \exp\left(\frac{3.6641 - \log\left(96\gamma\|R_k^\star\|^2\right)}{0.9820}\right). \tag{A.2}$$

This relationship allows us to choose a signature-specific concentration parameter to simulate new signatures, while controlling the resulting mean cosine errors across all signatures. We use the function $\beta\left(R_k^\star\right)$ to set the values of β_k for the γ-perturbed DGP described in "Simulating data generating processes".

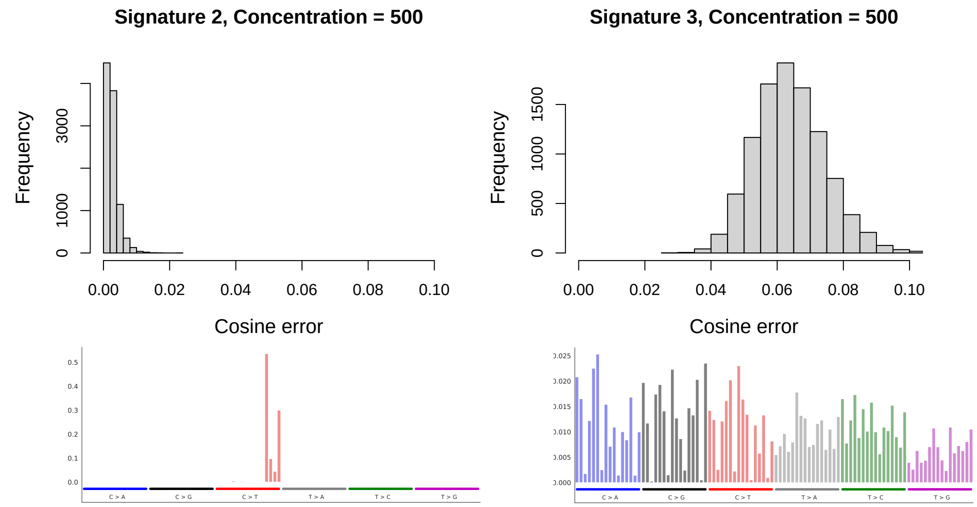

Fig A.1. Examples of simulating perturbed signatures. *(Top)* Distribution of cosine errors between $R_k^\star$ and 10,000 samples from Dirichlet$(\beta R_k^\star)$, where $\beta = 500$ and $R_k^\star$ is Signature 2 (left) or Signature 3 (right) from COSMIC v2. *(Bottom)* Mutation frequency profile for Signature 2 (left) and Signature 3 (right) from COSMIC v3.0.

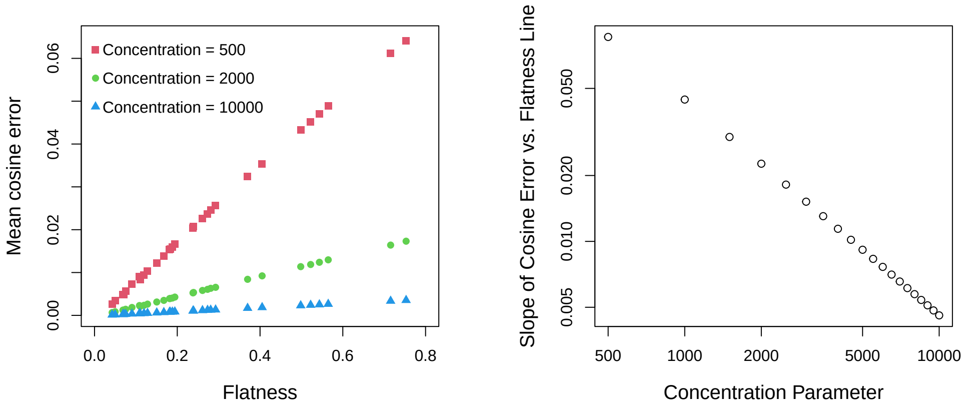

Fig A.2. Relationship between signature flatness and mean cosine error. *(1)* Strong linear relationship between flatness$(R_k^\star)$ and the mean cosine error between a sample from Dirichlet $(\beta R_k^\star)$ and $R_k^\star$ itself for several different values of β. *(2)* Strong linear relationship between the slope of the lines in Fig A.2A and the concentration parameter β on a log scale.

B Experimental details

For carrying out MCMC for the BayesPowerNMF model, we used pystan version 2.19.1.1. For each synthetic and real data set (excluding liver subsampling experiments), we initially consider candidate powers of 0.1, 0.2, 0.3, 0.4, 0.6, 0.8, and 1. For both real and synthetic melanoma data sets, all of these candidate powers resulted in inferring many spurious signatures in all pilot data sets, so we further considered candidate powers of 0.001, 0.002, 0.005, 0.01, 0.025, and 0.05. The powers selected via the BayesPowerNMF workflow are shown in Table B.1.

	Lung	Stomach	Skin	Ovary	Breast	Liver
PCAWG	0.4	0.2	0.01	0.6	0.2	0.6
well-specified	0.3	0.2	0.025	0.4	0.2	0.4
contaminated	0.4			0.3		0.4
perturbed	0.4			0.6		0.6
overdispersed	0.4			0.6		0.4

Table B.1. Powers selected via BayesPowerNMF workflow for our synthetic data sets (Simulations Results) and the real PCAWG data sets (Application Results).

For the synthetic liver subsampling experiments, we directly compute the power ξ_n used in BayesPowerNMF for each subsample size n per Equation 2.3 of Miller and Dunson [2] to maintain an equivalent level of "coarsening" from the standard posterior as using the power of 0.4 (selected via the full BayesPowerNMF workflow) for the full 326-sample cohort. That is, for $n = 20, 30, 50, 80, 120, 170, 230, 326$, we used powers of 0.916, 0.879, 0.813, 0.731, 0.644, 0.561, 0.486, and 0.400, respectively.

Runtime for the NMF model within BayesPowerNMF varied from 0.5-7 days per chain, with 10,000 burn-in and 10,000 sample iterations. Generally, smaller powers and cohorts had faster run time while misspecified data sets required longer runtime.

We used SigProfilerExtractor version 1.1.24. For both synthetic and real lung, ovary, and breast cancer data, we set $K_{\min} = 5$ and $K_{\max} = 15$; for skin cancer data, we set $K_{\min} = 5$ and $K_{\max} = 20$; and, for stomach and liver cancers, we set $K_{\min} = 7$ and $K_{\max} = 25$. For the synthetic liver subsampling experiments, we used $K_{min} = 2$ and $K_{max} = 20$ for all subsample sizes. For choosing the optimal K, we modify the algorithm presented in Islam et al. [3] to use a paired-samples Wilcoxon signed-rank test to compare the reconstruction error for each individual instead of a Mann-Whitney U test. This change led to choosing equal or larger K than the Mann-Whitney U test approach in all experiments.

We used the SignatureAnalyzer R functions as provided in Kim et al. [4]. We used the SigMoS R package as provided by Pelizzola et al. [5], using the same $K_{\min}$ and $K_{\max}$ values as for SigProfilerExtractor. COSMIC v2.0 and v3.0 SBS signatures were downloaded from https://cancer.sanger.ac.uk/signatures/downloads/.

C Additional empirical results

In this section, we provide additional context for the supplementary figures and tables.

Table C.1 and Table C.2 summarize the number of signatures inferred by each method (BayesPowerNMF, SigProfilerExtractor, SignatureAnalyzer, and SigMoS) for various cancer data sets. Table C.1 summarizes the results from six synthetic well-specified data sets while Table C.2 summarizes the misspecified data set; see "Simulated data" in the main text for a description of these data sets.

Fig C.1 shows example signatures inferred using SigMoS, corresponding to the results for BayesPowerNMF, SigProfilerExtractor, and SignatureAnalyzer shown in Fig 1 of the main text. We see that explicitly modeling overdispersed counts with SigMoS is not sufficient to account for more complex modes of misspecification, such as might be seen in real-world WGS data.

Fig C.2 is an example bubble plot that facilitates power selection in stage 4 of the BayesPowerNMF workflow as described in Fig 2 of the main text. Each panel corresponds to one simulated data set generated in stage 2, and each column corresponds to a different candidate power. Each circle represents one inferred signature, with position indicating the best match reference signature, color indicating recovery error, and size indicating the mean inferred loading. Any circles appearing above the red line indicate spurious signatures, and darker circles represent poor matches.

Fig C.3 shows that, across all 15 synthetic data sets, SigProfilerExtractor systematically misses more signatures with smaller ground truth mean loading (across the simulated patients in each data set) when compared to BayesPowerNMF or SignatureAnalyzer. Unlike BayesPowerNMF or SigProfilerExtractor, SignatureAnalyzer misses some signatures with very large loading. Here, a "missed" ground truth signature is defined as one without an optimal match in the inferred signature set.

Fig C.4 compares the recovery cosine error for each ground truth signature between BayesPowerNMF and SigProfilerExtractor across all 15 synthetic data sets. For a majority of ground truth signatures recovered by both methods, the recovery error is higher with SigProfilerExtractor than with BayesPowerNMF. We also observe that there are more ground truth signatures that are recovered by BayesPowerNMF but not SigProfilerExtractor (top edge of plot) than there are signatures recovered by SigProfilerExtractor but not BayesPowerNMF (right edge of plot).

Fig C.5 shows that the mean uncertainty in the posterior samples for each inferred signature from BayesPowerNMF is correlated with the recovery cosine error of the posterior mean for each inferred signature across the 15 synthetic data sets. We define the mean uncertainty in the posterior samples as the mean of the cosine error between each posterior sample for a signature and the posterior mean for that signature. The recovery error is computed between the posterior mean and its optimal match in COSMIC v2.

Fig C.6 extends the top panels of Fig 3 in the main text to also show the precision and recall curves of BayesPowerNMF using a power of $\xi = 1$ for signature recovery on the synthetic well-specified data sets. This approach reflects the standard Bayesian posterior for the model described in Eqs (1) to (5). Because these data sets are correctly specified according to the model, we expect that the standard posterior should perform well, and the BayesPowerNMF workflow using a power posterior may be more conservative. Indeed, we see here that BayesPowerNMF with $\xi = 1$ exhibits much better recall than the regular BayesPowerNMF result while maintaining comparable precision.

Fig C.8 shows the top five of ten signatures inferred by SigProfilerExtractor from

melanoma mutation counts data from the PCAWG project ("Application results"). This is the only one of the six real data sets where SigProfilerExtractor inferred more signatures than BayesPowerNMF. However, there appears to be duplication among some of these inferred signatures, which accordingly have some poor matches to the reference COSMIC v3. Of note, no SigProfilerExtractor solutions using $K = 2, \ldots, 20$ were considered optimal by the heuristic specified by Islam et al. [3]; we deemed $K = 10$ to be optimal as it was the only one with positive mean signature stability.

Table C.1. Number of signatures inferred in well-specified synthetic data. Mutational signature discovery results for simulated data, comparing SigProfilerExtractor, SignatureAnalyzer, and BayesPowerNMF on well-specified data for six cancer types. The number of COSMIC v2 signatures used to generate the data is in the "True" row. In each entry of the form X +Y *+Z* in the table, X = the number of estimated signatures that were matched to a ground truth signature with a cosine error of < 0.2, Y = the number of estimated signatures that were matched to a ground truth signature with a cosine error of ≥ 0.2, and Z = the number of estimated signatures that were matched to a COSMIC v2 signature that was not used to generate the data. Bold indicates the best performing method in each case. (SPE = SigProfilerExtractor; SA = SignatureAnalyzer; SM = SigMoS; BPN = BayesPowerNMF; $\text{BPN}_{\xi=1}$ = BayesPowerNMF with power of 1, which corresponds to the standard posterior.)

	Lung	Stomach	Skin	Ovary	Breast	Liver
True	13	21	14	12	12	21
SPE	7	11	6	7	**10**	9
SA	5 +4	5 +8 *+1*	6 +4 *+4*	5 +2	3 +5 *+2*	8 +6
SM	6	10 +1	4 +1 *+1*	7	6	14 +6 *+3*
BPN	**9**	10 +1	5 *+1*	7	7	15
$\text{BPN}_{\xi=1}$	9 +2	**14** +1	**13**	**10**	12 *+3*	**16** +1

Table C.2. Number of signatures inferred in well- and misspecified synthetic data. Same as Table C.1 but for both the well-specified and misspecified settings, for three cancer types.

	Lung (True = 13)				Ovary (True = 12)				Liver (True = 21)			
	SPE	SA	SM	BPN	SPE	SA	SM	BPN	SPE	SA	SM	BPN
well-specified	7	5 +4	6	**9**	**7**	5 +2	7	**7**	9	8 +6	14 +6 *+3*	**15**
α-contaminated	7	5 +4	6	**9**	5 +1	5 +2	6	**5**	9	7 +5	14 +7 *+4*	**15**
γ-perturbed	7	3 +7 *+1*	5	**10**	5 +1	5 +2	6 +1	**8**	9	10 +5	16 +4 *+2*	**17**
κ-overdispersed	7	4 +5	8 *+1*	**9**	5	5 +2	5	**9** +1	9	8 +7	16 +5 *+3*	**15**

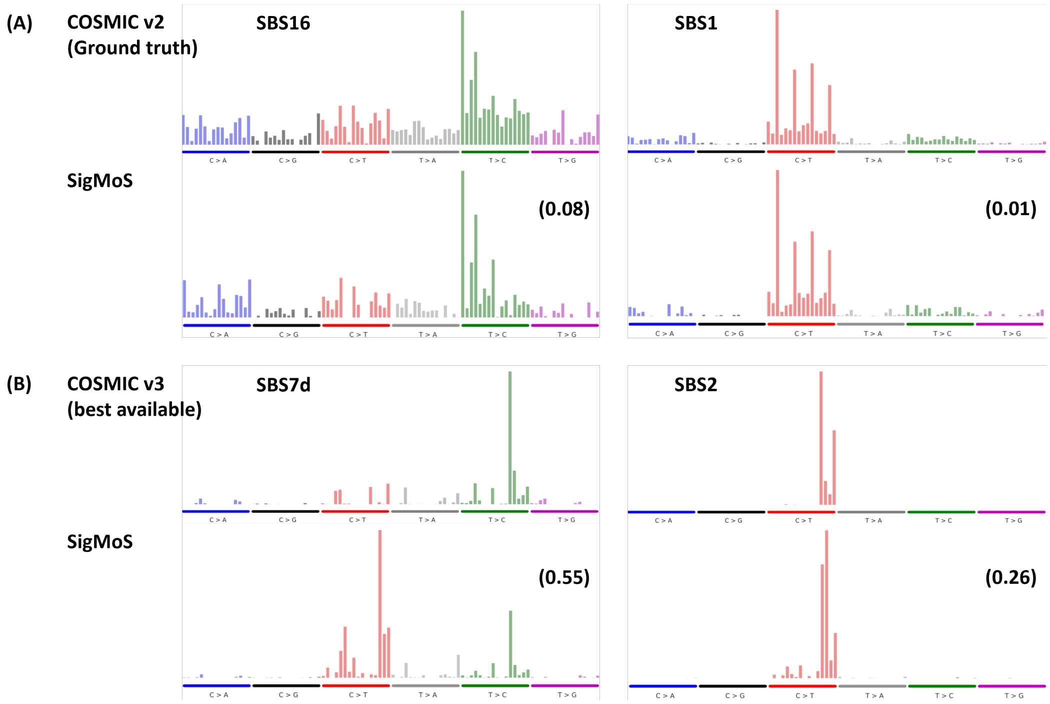

Fig C.1. Examples of SigMoS inferred signatures. Modeling overdispersed counts with SigMoS fails to account for alternate or more complex modes of misspecification. *(A)* Examples from simulated misspecified (γ-perturbed) liver cancer data. For two ground truth signatures used to generate the data (SBS16 and SBS1 from COSMIC v2, shown in the top row), we show the best-matching signatures inferred by SigMoS. The cosine error between the inferred and true signatures is in parentheses. *(B)* Examples from real melanoma data. Same as in (A), but comparing with the closest match to SBS7d and SBS2 from COSMIC v3. The SigMoS inferred signature that best matches SBS7d appears to be a linear combination with SBS7b. Refer to Fig 1 for the performance of BayesPowerNMF, SigProfilerExtractor, and SignatureAnalyzer.

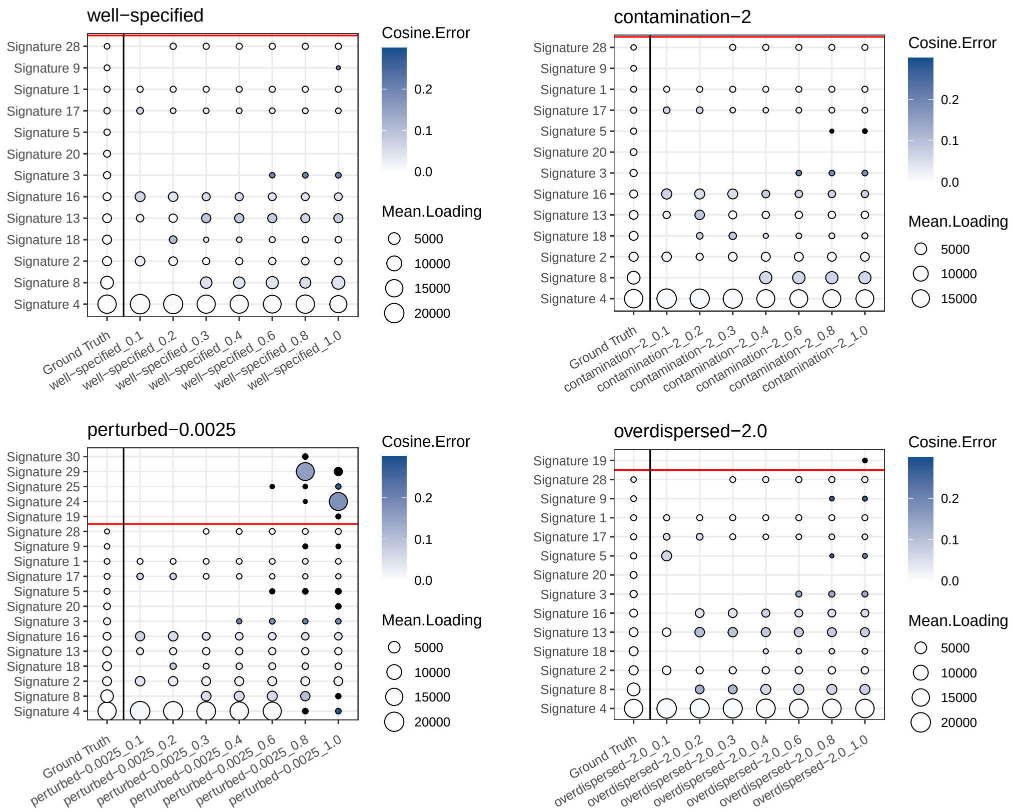

Fig C.2. Example of power selection. Bubble plots used to select the power ξ in stage 4 of the BayesPowerNMF workflow. Each panel corresponds to one simulated data set. See the description of Fig 4 for the interpretation of this type of plot.

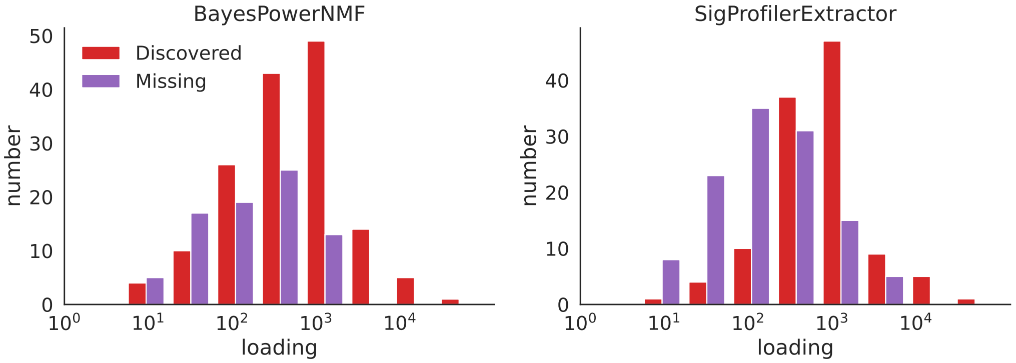

Fig C.3. Ground-truth loadings of discovered vs missed signatures by method. Histograms of ground-truth loadings for pilot signatures discovered and missed by BayesPowerNMF and SigProfilerExtractor across all simulated data sets. SigProfilerExtractor systematically misses many more signatures with small mean loading in the pilot data set.

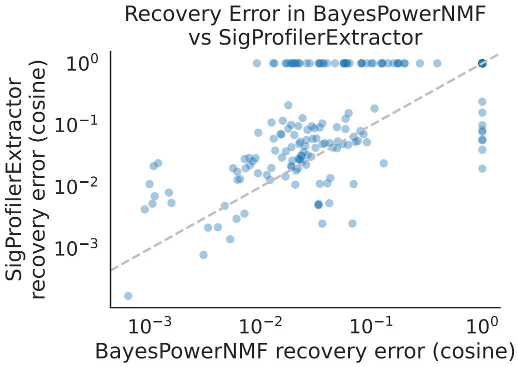

Fig C.4. Recovery error for SigProfilerExtractor versus BayesPowerNMF. For each of the 15 complete synthetic data sets (see "Simulated data"), for each ground truth signature, we plot the cosine error between the true signature and the signatures estimated by SigProfilerExtractor (y-axis) and BayesPowerNMF (x-axis); a cosine error of 1 indicates that no matching signature was inferred by that method. SigProfilerExtractor misses many of the signatures recovered by BayesPowerNMF (see dots along the top), and has higher cosine error for most of the signatures recovered by both methods (see dots above the diagonal).

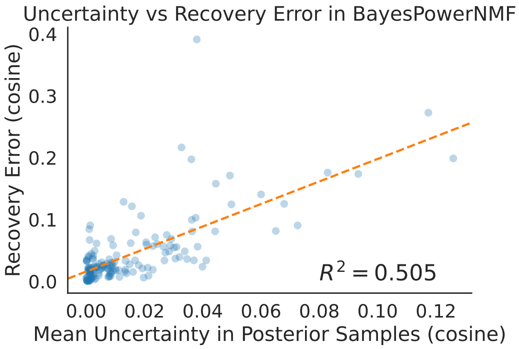

Fig C.5. Posterior uncertainty in BayesPowerNMF signatures. In simulations with BayesPowerNMF, the posterior uncertainty in each signature is correlated with the cosine error between the estimated signature and the ground truth signature. This indicates that the uncertainty quantification is providing meaningful information about the actual error. Here, uncertainty is defined as the mean cosine error between posterior samples and the posterior mean, for each signature.

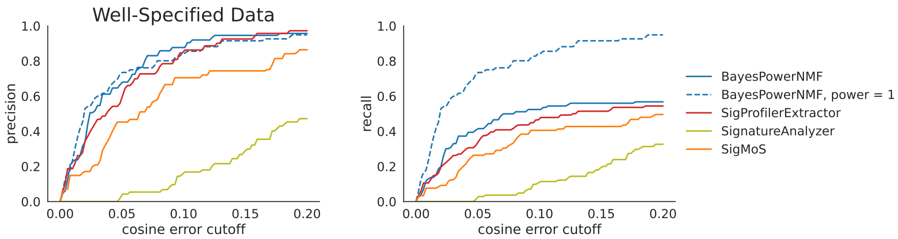

Fig C.6. Precision & recall for BayesPowerNMF with standard Bayesian posterior. Same as Fig 3 but also showing the precision and recall for the standard Bayesian posterior (blue dashed lines) for the same NMF model. This corresponds to BayesPowerNMF with a power of $\xi = 1$. When the model is correct (that is, in the well-specified case), the standard posterior exhibits higher recall than the power posterior, while maintaining comparable precision.

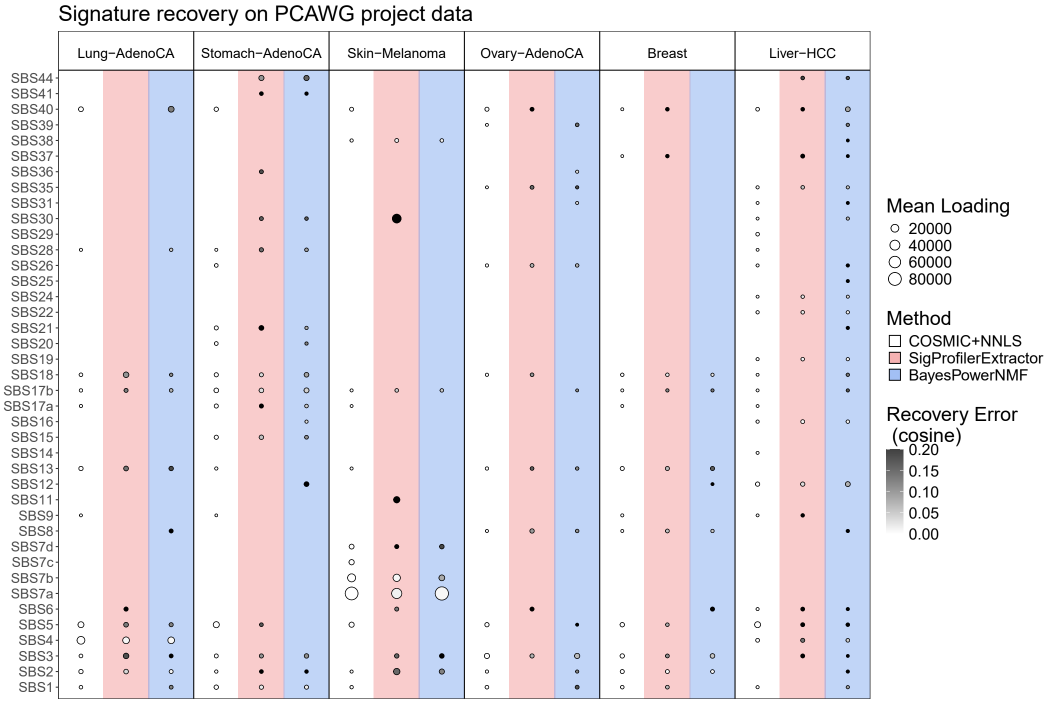

Fig C.7. Mean loading and cosine error between each estimated signature and its matching COSMIC v3 signature.
Results are shown for each method applied to the PCAWG data for each of six cancer types. See the description of Fig 4 for explanation of the plot. COSMIC+NNLS uses non-negative least squares to estimate loadings for the COSMIC v3 signatures.

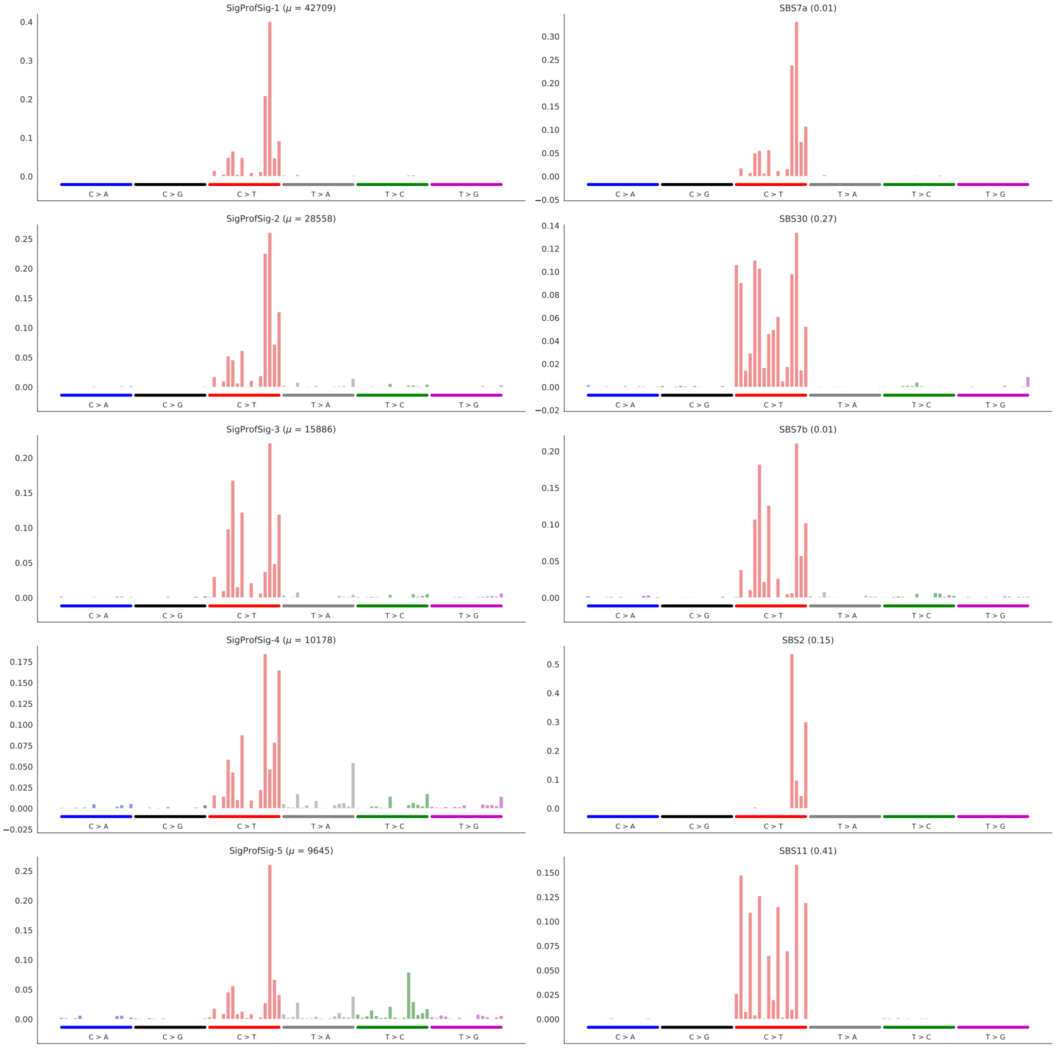

Fig C.8. Example of SigProfilerExtractor duplicating signatures. The top 5 out of 10 signatures inferred by SigProfilerExtractor from melanoma mutation counts from the PCAWG project (left) and their best match reference signatures from COSMIC v3 (right). There appears to be potential duplication between (a) SigProfSig-1, SigProfSig-2, and SigProfSig-5, and (b) SigProfSig-3 and SigProfSig-4, in the sense that these may be slightly perturbed versions of the same “true” signature.

References

1. Xue C, Zito A, Miller JW. Improved control of Dirichlet location and scale near the boundary. arXiv statME arXiv:241013050. 2024;.

2. Miller JW, Dunson DB. Robust Bayesian inference via coarsening. Journal of the American Statistical Association. 2018;114:1113–1125.

3. Islam SMA, Díaz-Gay M, Wu Y, et al. Uncovering novel mutational signatures by *de novo* extraction with SigProfilerExtractor. Cell Genomics. 2022;2:100179.

4. Kim J, Mouw KW, Polak P, et al. Somatic *ERCC2* mutations are associated with a distinct genomic signature in urothelial tumors. Nature Genetics. 2016;48:600–606.

5. Pelizzola M, Laursen R, Hobolth A. Model selection and robust inference of mutational signatures using Negative Binomial non-negative matrix factorization. BMC Bioinformatics. 2023;24:187. doi:10.1186/s12859-023-05304-1.
